# MARMoSET – Extracting Publication-ready Mass Spectrometry Metadata from RAW Files

**DOI:** 10.1074/mcp.TIR119.001505

**Published:** 2019-05-16

**Authors:** Marina Kiweler, Mario Looso, Johannes Graumann

**Affiliations:** ‡Bioinformatics Core Unit, Max Planck Institute for Heart and Lung Research, W.G. Kerckhoff Institute, Ludwigstr. 43, Bad Nauheim, Germany; §Scientific Service Group Biomolecular Mass Spectrometry, Max Planck Institute for Heart and Lung Research, W.G. Kerckhoff Institute, Ludwigstr. 43, Bad Nauheim, Germany; ¶The German Center for Cardiovascular Research (DZHK), Partner Site Rhine-Main

**Keywords:** Mass Spectrometry, Bioinformatics software, Quality control and metrics, Data standards, Bioinformatics

## Abstract

In the context of publishing data sets acquired by mass spectrometry or works based on such molecular screens, metadata documenting the instrument settings are of central importance to the evaluation and reproduction of results. A single experiment may be linked to hundreds of data acquisitions, which are frequently stored in proprietary file formats. Together with community-, repository-, as well as publisher-specific reporting standards, this state of affairs frequently leads to manual —and thus error prone—metadata extraction and formatting. Data extracted from a single file also often stand in for an entire file set, implying a risk for unreported parameter divergence. To support quality control and data reporting, the C# application MARMoSET extracts and reduces publication relevant metadata from Thermo Fischer Scientific RAW files. It is integrated with an R package for easy reporting. The tool is expected to be particularly useful to high throughput environments such as service facilities with large project numbers and/or sizes.

## Abstract (precis)

MARMoSET facilitates the extraction of meta data from Thermo Fischer Scientific RAW files and in the context of data sets often comprising hundreds of files reduces it to the smallest common set, suitable for quality control, reporting and publication.

## Graphical Abstract

**Figure:**
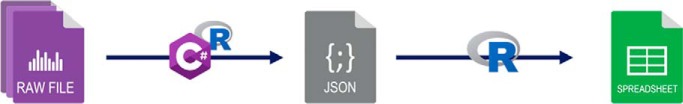

**Highlights**

- Automated metadata extraction from potentially large sets of mass spectrometric raw data.
- Reduction of extracted metadata into groups of shared parameter sets.
- Tabular representation for quality control, reporting and publication.

Aiming for evaluability and reproducibility of mass spectrometry based research and in parallel to the maturation of the field toward the acquisition of ever larger data sets, initiatives to standardize reporting of instrument settings and other relevant metadata have arisen in the community itself (1, 2), from the deposition requirements of public data repositories (3, 4), as well as have been launched by publishers and editors involved in the dissemination of mass spectrometric experiments (5, 6). The extraction and reporting of the metadata required remains a tedious process, especially given the facts that OMICS experiments frequently involve hundreds of data files and that data often resides in binary and/or proprietary file formats offering an excellent information to storage space ratio yet limiting ease of access.

One such example is the RAW file format produced by Thermo Fischer Scientific's (Bremen, Germany) mass spectrometers. Beyond the acquired spectral data, RAW files also contain instrument settings as metadata, which are required to evaluate and reproduce the results.

An obvious and common approach to extract this data is to manually open individual RAW files using the vendor-specific Xcalibur software and copy the required information. The tediousness and error prone nature of manual interaction with the individual file, however, frequently leads to the extraction of metadata describing an entire data set from a single file. When the data set encompasses hundreds of files acquired over a potentially long period, this implies a potential for undetected parameter drift with implications for laboratory-internal quality control, reporting and publication. In this context core facility laboratories carry a particularly large burden, as the sheer number of projects they handle further compounds the data access problem. In combination with publication requiring metadata reporting often years removed from data delivery to customers, the difficulty to extract parameters frequently implies “data archeology” from deep archive.

To the best of our knowledge no software exists to date to address the need for both simple reporting from large numbers of RAW files and metadata reduction to a consensus set of parameters. Using the vendor-provided application programming interface (API)^1^ RawFileReader (7) we create such a tool along with R (8) based infrastructure for the generation of tabular representations suitable for intra-laboratory quality control, data reporting, as well as supplemental material in publications.

## MATERIALS AND METHODS

### 

#### 

##### System Requirements

The C# (9) command line tool MARMoSET is running as 64-bit code on Microsoft Windows only. It was compiled in Visual Studio Community (Version 15.8.7, .net 4.7.03056) (10) for the .NET Framework 4.6.1. The accompanying R package is agnostic with respect to operating system and only requires a functional R installation, as well as the package dependences assertive (11), jsonlite (12), pathological (13), Rlist (14), stringi (15), and magrittr (16).

##### Implementation

The C# (9) application MARMoSET (https://github.molgen.mpg.de/loosolab/MARMoSET_C) extracts publication relevant metadata from Thermo Fischer Scientific RAW files as a JSON (17) file.

The RAW file format as accessed through the RawFileReader API provides multiple levels of metadata dependent on the system it was acquired on. A fixed header contains information like date, original filename, and sample information. The header is followed by a list containing instrument modules used and their respective methods as strings. The API additionally provides separate entry points for detector-associated data (such as ultraviolet spectrophotometry or mass spectrometry). MARMoSET currently only implements access to MS data, which beyond the acquired spectral data includes further instrument parameters and logs. Structure, string formatting, as well as location in the data structure of relevant metadata are specific for instrument classes. Using the “IRaw DataPlus” interface from the RawFileReader API, MARMoSET collects all relevant metadata from the divergent data structure.

In the context of liquid chromatography/mass spectrometry (LC/MS) using EASY-nLC ultra high-pressure liquid chromatography instruments (Thermo Fisher Scientific), LC parameters are available in the method strings and extracted and parsed by MARMoSET. Where chromatography instrument parameters beyond the EASY-nLC series are retrievable from the RAW file, future inclusion into the reporting infrastructure is expected to be straight forward and will be added as encountered.

**Fig. 1.**
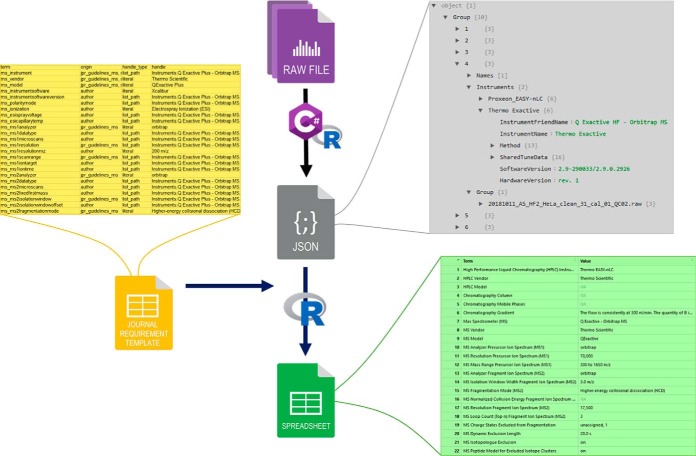
**Schematic representation of data processing by MARMoSET including excerpts of the data representations involved.**

Depending on whether it is provided with the path to a single RAW file or a directory, MARMoSET either acts on a single file or iterates over a collection of RAW files in a directory making use of parallelization dependent on the hardware resources available. In a first step, the metadata is gathered for each RAW file individually. In order to reduce data from many files into a minimal set of parameters describing the entire collection, the resulting data structures are hash code evaluated and sorted in a dictionary. This information is then used to sort RAW files into groups that share all relevant parameters. Finally, a JSON file is written, comprising a minimal set of parameter groups linked to the corresponding RAW file names (see Fig. 1).

To provide easy and intuitive handling of the structured data in the JSON file, we provide an R (8) package also named MARMoSET (https://github.molgen.mpg.de/loosolab/MARMoSET). It is designed to create compact and clear tables following predefined journal requirements such as MIAPE or JPR. Moreover, it supports individual selections of parameter sets in a few easy steps.

On Microsoft Windows, the included function “generate_json()” directly runs the C# command line tool from within R and captures its content into memory. Alternatively, externally generated JSON files may be read as well. In-memory data is reformatted by list flattening into a “data.frame” (function “flatten_json()”).Based on a term-matching Table included in the package, the function “match_terms()” extracts and arranges a subset of parameters from the total metadata set in the flattened JSON file for all given parameter groups and generates a table reduced to journal specific requirements. These tables may then be exported ready to upload in the form of tab delimited txt files and MS excel tables by using the “save_all_groups()” function. The included term-matching table can easily be adapted by the user to include further metadata entities or to design individual reporting styles. It is noteworthy that the same toolkit employed here has also been choosen by Trachsel et al (2018) (17) to facilitate analysis of spectrum-level metadata.

##### Exemplary Workflow (Windows)

In a first step and from within a functional R environment (see https://cloud.r-project.org/ for installation instructions), the MARMoSET R package is installed from the github repository using the package remotes (18), which may be achieved by calling “install.packages(“remotes”); remotes::install_github(“loosolab/MARMoSET,” host = “github.molgen.mpg.de/api/v3”).” After loading MARMoSET with “library(MARMoSET),” a JSON object containing the metadata of grouped RAW files may be created by executing “json <- generate_json(“<PATH-TO-RAW-FILES-DIRECTORY>”)” and prepared for downstream processing using “flat_json <- flatten_json(json),” A reporting guideline- (“MIAPE” in this example) and instrument-specific filter is generated by calling “term_matching_table <- create_term_matching_table(instrument_list = c(“Thermo EASY-nLC”, “Q Exactive - Orbitrap_MS”), origin_key = “miape”)” and applied to the JSON object using “vector_of_group_tables <- match_terms(flat_json, term_matching_table).” Finally, a tab delimited text file representation may be saved using “save_all_groups(vector_of_group_tables, output-path).”

Further use cases and more detailed instructions may be found on the top-level README page of the github repository (https://github.molgen.mpg.de/loosolab/MARMoSET), as well as through R's help system (*e.g.* by calling '?generate_json') (19).

## RESULTS AND DISCUSSION

A combination of an ever-increasing number of raw data files per mass spectrometry based experiment and a strong push for the standardized reporting of metadata for evaluation and reproducibility has rendered metadata extraction and its reduction into the smallest common parameter set a common need within the community. To the best of our knowledge, however, no tool simplifying metadata extraction currently exists. With the MARMoSET suite of tools presented here we fill that gap for mass spectrometric data acquired by Thermo Fisher Scientific's instruments, combining outputs geared toward machine readability (JSON), as well as human consumption (tab delimited text and MS excel). The resulting information is suited for documentation and reporting, publication, as well as operations oversight and expected to be particularly helpful in the context of environments implying high throughput acquisition of mass spectrometric data, such as core facility laboratories.

In conclusion, MARMoSET offers tools for the simple extraction of metadata from RAW files. With the intend to particularly serve high throughput data acquisition environments, the tool enables the straightforward generation of small and clear tables containing just the metadata or parameter information needed. MARMoSET is designed for flexible adaption on individual laboratory's needs.

## Footnotes

^1^ The abbreviation used is:

API: application programming interface.
